# Blinding Trachoma: A Disease of Poverty

**DOI:** 10.1371/journal.pmed.0010044

**Published:** 2004-11-30

**Authors:** Pashtoon M Kasi, Ahmed I Gilani, Khabir Ahmad, Naveed Z Janjua

## Abstract

Trachoma accounts for 15% of blindness worldwide, affecting the world's poorest communities. How can the disease be controlled?

Trachoma is almost exclusively a disease of poor families and communities living in developing countries. It accounts for 15% of blindness worldwide—around 6 million people [1]. Although the disease is avoidable, it continues to blind. With so few voices speaking out on behalf of people affected by trachoma, it remains a neglected public health issue.

## Epidemiology

Trachoma, a chronic keratoconjunctivitis, is caused by episodes of infection with Chlamydia trachomatis, an obligate intracellular bacterium. Only serovars A, B, Ba, and C are implicated in trachoma.

**Figure 1 pmed-0010044-g001:**
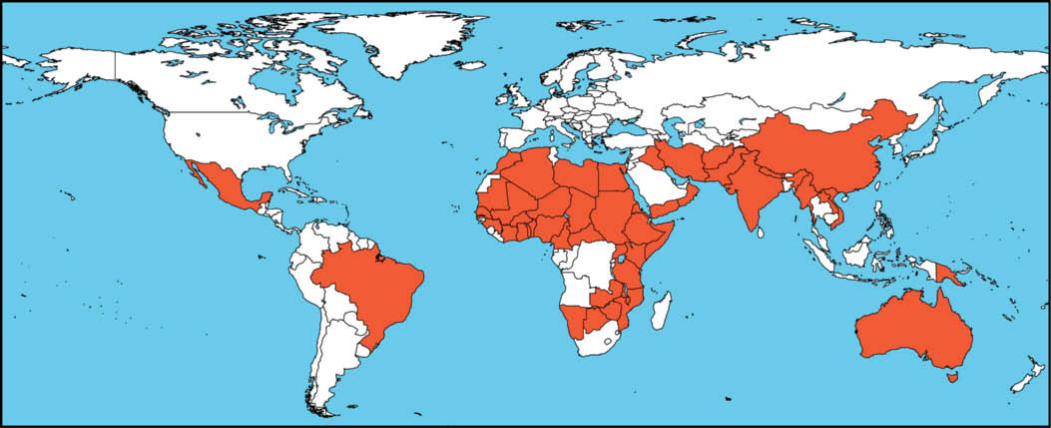
The Worldwide Distribution of Trachoma (Map: Silvio Mariotti/WHO)

Trachoma is the second leading cause of blindness worldwide [1]. According to the World Health Organization, currently 84 million people, mostly children, have active disease, and another 7.6 million people have trichiasis—a stage of trachoma in which the upper eyelid turns inward and one or more eyelashes rub against the eyeball [2]. An estimated 10% of the world's population lives in endemic areas and is at risk of developing trachoma. Global loss of productivity related to impaired vision and blindness from trachoma is thought to be as high as $US 5.3 billion annually [3]. More than 55 countries have been identified as endemic for trachoma, most of them in Africa and Asia (Figure 1) [4].[Fig pmed-0010044-g001]

Humankind has known trachoma since antiquity. Ibn-e-Isa, an Arab physician, was the first person to describe the different stages of trachoma and noted trichiasis as one of its sequelae. So prevalent was the disease not so long ago that trachoma hospitals were established in many parts of Europe and America [5]. The disease then declined dramatically in what is now called the developed world, mainly because of socioeconomic development [4].

**Figure 2:**
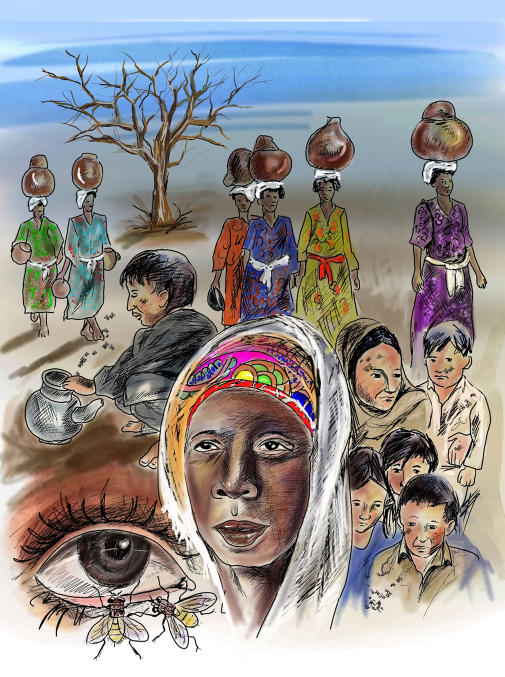
A Typical Community in Which Trachoma Is Endemic Some of the factors linked with the continued presence of the disease in affected communities are lack of access to water, overcrowding, lack of facial hygiene, eye-seeking bazaar flies, and open latrines. (Illustration: Aslam Bashir, Aga Khan University)

Transmission occurs from eye to eye via hands, clothing, and other fomites. Flies have been identified as a major vector for the infection's spread [6]. The presence of open latrines favors the vector population (Figure 2). Factors associated with trachoma include the extent to which the water supply is limited, the distance from the water source, the amount of water used for washing purposes, and overcrowding [7]. One case-control study in a Gambian village compared water use in 18 families having one or more active trachoma cases among the children with that in 16 trachoma-free families in the same village. The families with trachoma were found to use significantly less water per person per day for washing children than did the control group [8].

The disease tends to cluster in certain communities within a village and certain families within a neighborhood. Women, especially in rural areas, are affected twice as often as men [9].

## Clinical Manifestations and Grading

**Figure 3:**
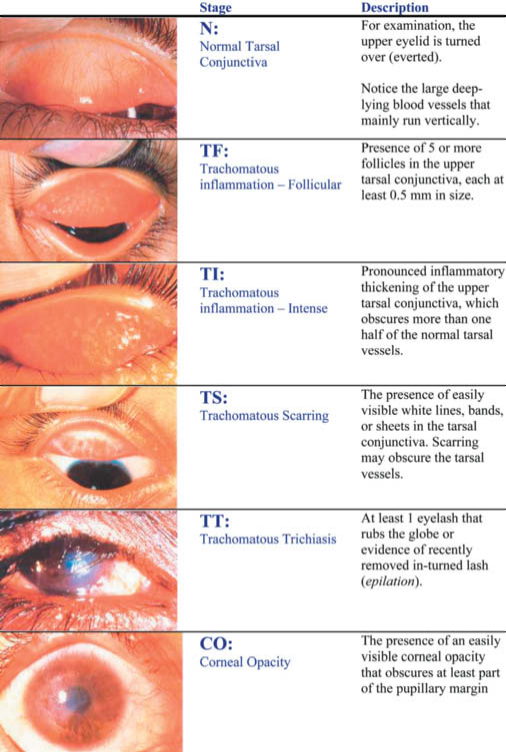
WHO Simplified Grading System: A Guide for the Assessment of Trachoma (Photos: from [11], with permission from WHO)

Trachoma initially presents in childhood as red eye—itching, redness, and pain. The essential lesion is a trachomatous follicle (lymphoid cell aggregate) occurring typically in the upper tarsal conjunctiva (Figure 3). The roughened appearance of the upper tarsal conjunctiva gives the disease its name (trachoma is the Greek word for “rough”). Trachomatous involvement of the cornea manifests itself initially as superficial keratitis. At a later stage, pannus formation (new vessel growth) may occur over the margin of the cornea, usually limited to the upper half.

In a subgroup of individuals, fibrosis occurs because of repeated infections, resulting in scarring of the conjunctiva (scarring trachoma). In scarring trachoma, the upper eyelid is shortened and distorted (entropion) and the lashes abrade the eye (trichiasis). Blindness results from corneal opacification, which is related to the degree of entropion or trichiasis [10].

Based on the presence or absence of some of the key signs of the disease, WHO has developed a simplified grading system for the assessment of trachoma (Figure 3) [11,12]. The system can easily be used by non-specialists, after appropriate training, for the assessment of disease at the community level.

Herbert's pits (healed follicles in the superior limbus) and Arlt's line (a horizontal scar on the upper tarsal conjunctiva) are two other classical features of the disease.

## Managing Trachoma: The SAFE Strategy

WHO currently recommends the “SAFE” strategy for the management of trachoma: Surgery for trichiasis, Antibiotics for active disease, Facial hygiene, Environmental improvement to reduce the transmission of the disease [13,14,15].

### Surgery.

People with trachomatous trichiasis are at risk of blindness, and so treating these people is the first priority for the SAFE strategy. An evidence-based review of the SAFE strategy found that trichiasis surgery can alleviate discomfort and improve vision, though the evidence is less clear on whether such surgery prevents corneal opacification [14]. The review authors suggested that a protective effect of surgery against opacification is likely.

There are different types of surgical procedures to correct trachomatous trichiasis [16]. Their high costs and the lack of surgical expertise in endemic regions, however, restrict the use of many of these as public health interventions. On the basis of a controlled trial by Reacher and colleagues [16], WHO recommends the bilamellar tarsal rotation procedure as the preferred technique; it is easy to perform and easy to learn [17]. Surgical effectiveness is defined in terms of recurrence of trichiasis; in the controlled trial, bilamellar tarsal rotation produced a recurrence rate of around 20% at follow-up 9–21 months after surgery, while other procedures saw 60% of patients with recurrence of trichiasis in the same period [16]. In several countries, different levels of health staff, including nurses and ophthalmic assistants, have been trained to perform the bilamellar tarsal rotation procedure.

In addition to recurrence, there are other problems with the surgical approach to managing trachoma. It cannot correct all the complications, such as dry eyes. Even more important, and the main obstacle to preventing blindness from trachoma, is the low rate of uptake of surgery by communities with trachomatous trichiasis [14]. Barriers to uptake include distance to travel to surgery, perceived cost of the operation, child care duties, and lack of awareness about the treatment [14]. In Tanzania, less than a fifth of women with trichiasis opted for surgery, even when it was offered for free [18].

Offering surgery at the community level, rather than in distant medical facilities, is one strategy that could reduce travel times and costs and increase uptake. A cluster randomized controlled trial of village-based surgery versus health-center-based surgery in Gambia found a significantly higher uptake rate with the village-based service [19].

### Antibiotics.

The use of antibiotics aims to treat active infection and eliminate the reservoir. WHO currently recommends two regimens for the treatment of trachoma in endemic regions. These are 1% topical tetracycline ointment (twice daily for six weeks) or a single dose of oral azithromycin (1 g in adults and 20 mg/kg in children) [20].

Although antibiotics are a cornerstone of the SAFE strategy, clinical trials of antibiotics versus control (no treatment, placebo, or vitamin tablets) have produced conflicting results and are difficult to pool because of their heterogeneity. A recent Cochrane systematic review concluded “there is some evidence that antibiotics reduce active trachoma but results are not consistent and cannot be pooled” [20]. The review also found that “oral treatment is neither more nor less effective than topical treatment” [20].

Several questions remain about the use of antibiotics, such as who should receive them and how often. Lietman and colleagues have developed a mathematical model of frequency of treatment that uses available epidemiological data from a variety of countries [21]. Based on their model, they recommend that in areas where trachoma is moderately prevalent (less than 35% of children with active infection), it should be treated annually, but in hyperendemic areas (more than 50% of children with active infection), it should be treated biannually. Such models, however, need to be validated by well-designed clinical trials.

### Facial hygiene.

Good facial hygiene aims to reduce transmission, the risk of autoinfection in a community, and the risk of attracting flies [13,15]. Many cross-sectional surveys have shown that children with clean faces are less likely to have trachoma, and are less likely to have severe trachoma [14]. A recent study in Mali found dirtiness of the face to be the most important risk factor associated with trachoma [22].

A Cochrane systematic review found evidence that face washing combined with topical tetracycline can be effective in reducing severe active trachoma [23]. However, the evidence does not support a beneficial effect of face washing alone or in combination with topical tetracycline in reducing non-severe active trachoma [23].

Interventions aimed at promoting facial hygiene have not yielded expected results in all settings, as behavioral change is not always readily achievable.

### Environmental improvement.

This component of the SAFE strategy also aims to reduce transmission of trachoma by eliminating or reducing its risk factors, some of which are ubiquitous while others are specific to a region. Improving access to water is a key element. Other measures, such as provision of latrines to reduce the fly population, have also been found effective in reducing transmission [6]. Such environmental improvements will also provide other health benefits to a community, such as reduction in the incidence of diarrhea.

As mentioned previously, there is an important association between water and trachoma—though the association is not a simple one. The distance to the water source constrains the amount of water used for hygiene practices. Improving access to water on its own, however, may not be enough. In the case-control study in Gambia, families with trachoma used less water per person per day for washing children than families without the disease, regardless of the amount of water available [8]. In other words, interventions aimed at increasing the availability of water should also promote its appropriate use. Getting community “buy in” for these interventions is important.

## Why Is Trachoma So Neglected?

Trachoma is a disease of poor, underprivileged, and socioeconomically disadvantaged communities. It affects people who have little or no say in public decision making [24]. Investing in trachoma may sometimes mean compromising on other important issues. Many countries in which trachoma is endemic are also marred by regional conflicts, civil wars, and widespread corruption. Scarce resources are being spent on arms and debt servicing. These countries often lack the political commitment needed to fight against the disease. In addition, there is a lack of commitment by international donors.

Still, there is some room for optimism, given WHO's vision of the global elimination of trachoma by the year 2020 and the efforts of the International Trachoma Initiative and other non-governmental organizations. The implementation of the SAFE strategy to eliminate blinding trachoma has already proven effective in several countries [25]. Many countries have already started trying to eliminate trachoma themselves.

## Future Directions

Although the new initiatives in trachoma control are encouraging, trachoma elimination programs clearly need to be extended to many more countries. In addition, there are three crucial steps that still need to be undertaken if blindness from trachoma is to be eliminated.

First, there needs to be more emphasis on the “F” and “E” components of the SAFE strategy. Antibiotics and surgery alone will not eliminate trachoma; work also needs to be done to eliminate the risk factors and decrease the transmission of the disease in affected communities. Such primary prevention is more likely to have a sustainable impact but requires a prolonged effort and investment [15,24]. Because elimination of trachoma requires improvement in education and hygiene practices, improved accessibility to water, and economic development of endemic regions, collaboration among departments and ministries is vital. An example of such collaboration is the recent involvement of the Water Supply and Sanitation Collaborative Council (www.wsscc.org) in trachoma control efforts. Similar partnerships need to be strengthened [25]. Socioeconomic development must be at the heart of control efforts—trachoma was eradicated from much of the developed world even before the advent of antibiotic programs for trachoma, and much of this eradication was attributable to socioeconomic development [26].

Second, research into different aspects of the disease should continue. Work on a vaccine for trachoma, although not successful thus far, should receive more attention [10]. Future research should look at risk factors for trachoma in diverse communities and at barriers to implementation of the SAFE strategy.

Third, awareness about the disease and the SAFE strategy need to be promoted globally. At the same time, local, cost-effective solutions to trachoma need to be encouraged. Provision of pit latrines to reduce fly populations is just one such measure [6].

Unless these steps are taken, trachoma will continue to be a major cause of blindness in communities in the developing world [24].

## Abbreviation

WHO: World Health Organization
